# The role and possible molecular mechanism of valproic acid in the growth of MCF-7 breast cancer cells

**DOI:** 10.3325/cmj.2017.58.349

**Published:** 2017-10

**Authors:** Xiao-jie Ma, Yun-shan Wang, Wei-ping Gu, Xia Zhao

**Affiliations:** 1 Department of Otorhinolaryngology, Qilu Hospital of Shandong University, Jinan, Shandong, China; 2 Medical Research & Laboratory Diagnostic Center, Jinan Center Hospital Affiliated to Shandong University, Jinan, Shandong, China; 3 Department of Pharmacy, Shandong Provincial Qianfoshan Hospital, Shandong University, Jinan, Shandong, China; 4 Department of Laboratory Medicine, Shandong Provincial Qianfoshan Hospital, Shandong University, Jinan, Shandong, China

## Abstract

**Aim:**

To investigate the role of valproic acid (VPA), a class I selective histone deacetylase inhibitor, on Michigan Cancer Foundation (MCF)-7 breast cancer cells, named and explore its possible molecular mechanism.

**Methods:**

MCF-7 cells were cultured with sodium valproate (0. 5-4.0 mmol/L) for 24 h, 48 h, and 72 h *in vitro*, respectively. The cell viability, apoptosis, and cell cycle were examined. The activities and protein expressions of caspase-3, caspase-8, and caspase-9 were subsequently assayed. Finally, mRNA and protein expressions of cyclin A, cyclin D1, cyclin E, and p21 were analyzed.

**Results:**

Sodium valproate suppressed MCF-7 cell growth, induced cell apoptosis, and arrested G1 phase in a time- and concentration- dependent manner, with the relative cell viabilities decreased, cell apoptosis ratios increased, and percentage of G1 phase enhanced (*P* < 0.05). Increased activity of caspase-3 and caspase-9, but not caspase-8, and increased protein levels were found under sodium valproate (2.0 mmol/L, 48h). P21 was up-regulated and cyclin D1 was down-regulated at both mRNA and protein levels under sodium valproate (2.0 mmol/L, 48h)(*P* < 0.05), although cyclin E and cyclin A remained changed.

**Conclusion:**

These results indicate that VPA can suppress the growth of breast cancer MCF-7 cells by inducing apoptosis and arresting G1 phase. Intrinsic apoptotic pathway is dominant for VPA-induced apoptosis. For G1 phase arrest, p21 up-regulation and down-regulation of cyclin D1 may be the main molecular mechanism.

Histone acetylation is dynamically regulated by histone acetyltransferase (HAT) and histone deacetylase (HDAC). HATs lead to the relaxation of chromatin structures and genes transcriptional activation, whereas HDACs are associated with chromatin condensation and transcriptional silence (1,2). Alterations in eukaryotic chromatin structures caused by acetylation of the core histone N-terminal domains seem to play a central role in the regulation of gene transcription. Recent studies have demonstrated the correlation between histone acetylation or deacetylation and the genesis and development of some types of tumors (3-5). Thus, a new approach to tumor therapy emerged: activation of HATs and/or suppression of HDACs.

Histone deacetylase inhibitors (HDACIs), such as sodium butyrate (6), apicidin (7), and trichostatin A (8), showed antitumor functions, including cell growth inhibition, apoptosis induction, tumor cell re-differentiation stimulation, and cell cycle arrest induction in G1 phase. Valproic acid (VPA), which has been used widely as an anticonvulsant for over 20 years and has been well known for its low noxious and prolonged effectiveness (9), was demonstrated as a class I selective histone deacetylase inhibitor (10). Multiple antiproliferative effects of VPA resulting from inhibition of deacetylase activity were reported in various malignancies, such as human choriocarcinoma (11), thyroid cancer (12), hepatic cancer (13), pancreatic cancer (14), breast cancer (15), and so on. Various gene expression changes were further proved under inhibition of deacetylase activity, including up-regulation of multiple cyclin-dependent kinase inhibitors and down-regulation of cyclins, nuclear receptors, and p53 (16). A similar study in oral squamous cell carcinoma (OSCC) showed that treatment with VPA increased the cells distributed in G1 phase and reduced cells in the S phase of human tongue squamous cell carcinoma cells CAL-27 (17). However, further studies for VPA’s anti-tumor function and molecular mechanism are still needed, especially in breast cancer (18,19), which occurs at high incidence in China.

The aim of the present study was to investigate the role of VPA on cultured MCF-7 cells, a typical breast cancer cell line, and further explore its possible molecular mechanism, particularly in cell apoptosis and cell cycle. Our hypothesis was that VPA could inhibit the growth of Michigan Cancer Foundation (MCF)-7 by inducing cell apoptosis and affecting its cell cycle. Our data showed that VPA suppressed MCF-7 growth, induced apoptosis, and arrested G1 phase. Intrinsic apoptotic pathway is dominant in VPA-induced apoptosis, and p21 up-regulation and down-regulation of cyclin D1 may be the main molecular mechanism for G1 phase arrest.

## MATERIALS AND METHODS

### Reagents

The human breast cancer cell line MCF-7 was obtained from the American Type Culture Collection (ATCC). Gibco media RPMI 1640 and fetal bovine serum (FBS) were used (Invitrogen Co., Carlsbad, CA, USA). Anti-caspase-3, cleaved caspase-3, caspase-9, cleaved caspase-9, caspase-8, cleaved caspase-8, and p21Waf/cip1 [below as p21) antibodies were obtained from Cell Signaling Technology (Boston, MA, USA). Anti-cyclin A, cyclin D1, cyclin E, and β-actin antibodies were purchased from Santa Cruz Biotechnology (Cambridge, UK). Sodium valproate, Cell Counting Kit-8 (CCK-8), cell apoptosis related reagents, cell cycle detection reagents, and all other reagents used in the present study were all Sigma products (St. Louis, MO, USA).

### Cell culture and VPA exposure treatment

MCF-7 cells (1.0 × 10^5^/mL) were cultured in 10% FBS-1640 medium, with 100 U/mL penicillin and 100 mg streptomycin at 37şC in a humidified atmosphere composed of 95% air and 5% CO_2_. For VPA exposure experiment, cells were divided into control and VPA treatment groups. The control cells were cultured as common and equal amounts of phosphate buffered saline (PBS) were added to both groups of cells. For VPA treatment groups, different concentrations of sodium valproate (0.5, 1.0, 1.5, 2.0, 2.5, 3.0, 3.5, and 4.0 mmol/L) were added into the medium when cells reached 70%-80% of confluence, with different exposure times (24 h, 48 h, and 72 h).

### The relative cell viability detection under CCK-8

Cells (1.0 × 10^5^/mL) were subcultured in a 96-well cell culture cluster (Corning Inc., Corning, NY, USA) (100μL/well), and each sodium valproate concentration group was set to four replicates. CCK-8 (10 μL) was added into each well 1-4 h before the indicated time points. After 1-4 h of incubation at 37şC, the optical density (OD) values were measured at 535 nm using an ELISA reader (Multiskan GO, Thermo Scientific, MA, USA). Relative cell viability was calculated according to the following formula:

Relative cell viability (%) = (ODVPA-ODblank) / (ODcontrol-ODblank) × 100%

### Cell apoptosis analysis based on flow cytometry

Cells were trypsinized and collected at the indicated time points as common. As described in the instructions, cells (1.0 × 10^6^/group) were suspended in 200 μL binding buffer containing Annexin V/FITC (5 μL) and PI (10 μL) for 15 minutes at room temperature. Then binding buffer (300 μL) was added into the tube for further detection by flow cytometry (Becton Dickinson, Mountain View, CA, USA). All experiments were performed in triplicate.

### Assessment of cell cycle

Cells were trypsinized and collected at the indicated time points as common. After washing twice by cool PBS, cells (1.0 × 10^6^/group) were fixed in 75% frozen ethanol at -20şC for 1 hour. Cells were then suspended in 200-500 μL cool PBS and, after 20 μL of solution A were added, incubated in a water bath at 37 şC for 30 minutes as described in the instructions. Then 400 μL of solution B were added, and cells were incubated for 30-60 minutes at 4şC, avoiding light. Results were analyzed by flow cytometry (FACSCanto^TM^ II, Becton Dickinson, NJ, USA). All experiments were performed in triplicate. Further cell cycle analysis was conducted based on ModFit LT software.

### Reverse transcription-polymerase chain reaction (RT-PCR) detection

Total RNA was extracted using TRIzol (Invitrogen) as common (20). Reverse transcription was performed using the cDNA synthesis kit in a final volume of 20 μL containing 3 μg total RNA, and 1 μL Oligo (dt) 18 Primer (0.5 μg/μL). PCR was conducted according to the instructions of Takara TaqTM. The general amplication conditions were 94°C, 5 minutes; 94°C, 45 seconds; 56 ~ 61.5°C, 45 seconds; 72°C, 45 seconds; 30-35 cycles. Proper volume of the amplified product was subjected to 1% agarose gel for electrophoresis. All experiments were performed in triplicate. The relative optic density (OD) ratio was calculated with the NIH ImageJ software (US National Institutes of Health, Bethesda, MA, USA) in comparison with β-actin. The primers were designed using Primer 5.0 Software (Table 1).

**Table 1:** The sequences of primers for reverse transcription-polymerase chain reaction (RT-PCR)

Gene	Forward-primer (5′→3′)	Reverse-primer (5′→3′)
cyclin A	ACCCCTTAAGGATCTTCCTG	TCCAGGGTATATCCAGTCTTTCG
cyclin D1	GGATGCTGGAGGTCTGCGAGGAAC	GAGAGGAAGCGTGTGAGGCGGTAG
cyclin E	ATACAGACCCACAGAGACAG	TGCCATCCA CAG AAATACTT
p21	CAGGCGACAGCAGAGGAAGA	GGGCGGCCA GGGTATGTAC
β-actin	CTCACCCTGAAGTACCCCATCG	CTTGCTGATCCA CATCTGCTGG

### Western blot analysis

Total protein was extracted using radio immunoprecipitation assay (RIPA) and was quantified using a BCA kit (Sigma) according to the manufacturer’s instructions. Samples (80 μg of total protein each) were used in Western blot analysis with the first antibodies (caspase-3 1:1000, cleaved caspase-3 1:1000, caspase-9 1:1000, cleaved caspase-9 1:1000, caspase-8 1:1000, and cleaved caspase-8 1:1000, cyclin A 1:200, cyclin D1 1:200, cyclin E 1:200, p21 1:1000, and β-actin 1:2000). The relative OD ratio was calculated with the NIH ImageJ software in comparison with β-actin from three experiments.

### Statistical analysis

Data were presented as mean ± standard deviation (SD). Normality test was performed using Kolmogorov-Smirnov test. One-way analysis of variance (ANOVA) was applied to analyze these data. For post hoc test, Student-Newman-Keuls method was chosen. Pearson correlation analysis was applied to analyze the correlation between sodium valproate exposure time or concentration and the relative cell viabilities of MCF-7. *P* values less than 0.05 were considered statistically significant. Statistical calculations were performed using the SPSS 16.0 software package (SPSS Inc., Chicago, IL, USA).

## RESULTS

### VPA exposure inhibited the growth of MCF-7cells

**Figure 1:**
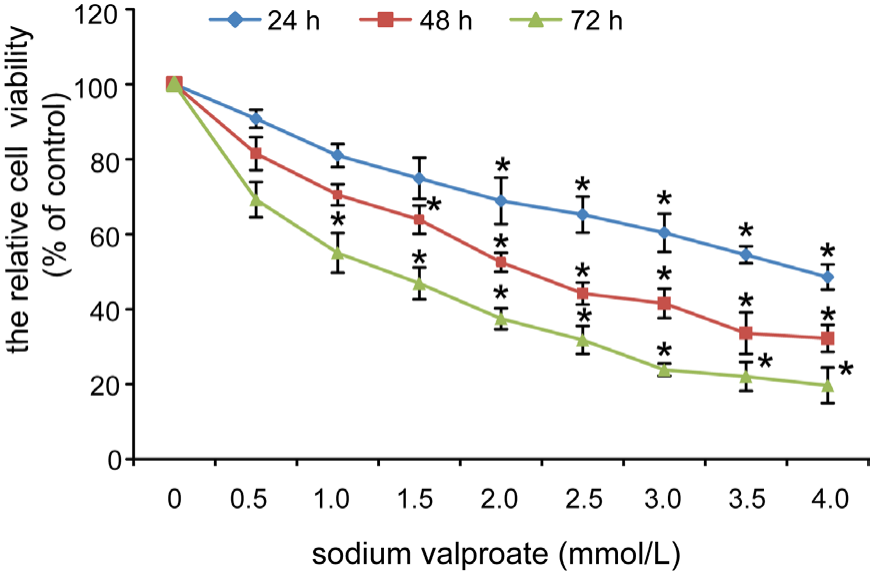
Valproic acid (VPA) exposure decreased the relative cell viabilities of Michigan Cancer Foundation (MCF)-7 in a time- and concentration-dependent manner.

The relative cell viabilities of MCF-7 exposed to sodium valproate (0.5 ~ 4.0 mmol/L) for 24 h, 48 h, and 72 h decreased in a concentration- and time- dependent manner (Figure 1). Taken the relative cell viabilities of control MCF-7 (sodium valproate 0 mmol/L) as 100%, the relative cell viabilities of MCF-7 under sodium valproate (2.0 mmol/L, 24 h) were 68.9 ± 6.2% and significantly decreased (*P* < 0.05). For 48 h, the statistical decrease of relative cell viabilities of MCF-7 could be found from sodium valproate (1.5 mmol/L), and from sodium valproate (1.0 mmol/L) for 72 h exposure (*P* < 0.05). Pearson correlation analysis showed that sodium valproate inhibited the growth of MCF-7 cells in a concentration- and time-dependent manner (r = 0.825 and r = 0.416, respectively, *P* < 0.001 for both).

### VPA exposure induced apoptosis of MCF-7 cells under flow cytometry

**Figure 2:**
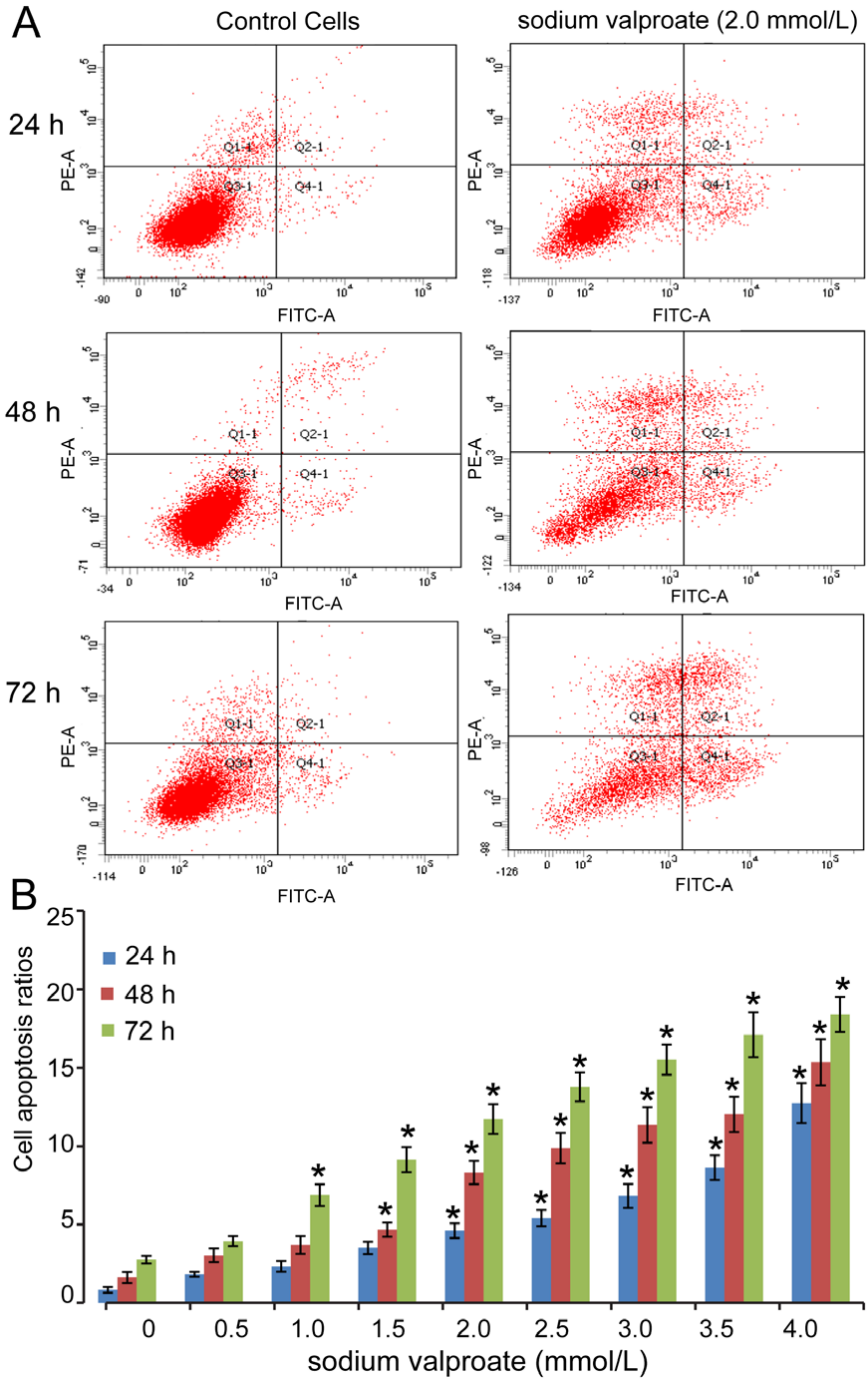
Cell apoptosis was induced under valproic acid (VPA). **A**. Flow cytometry showed increased apoptosis of Michigan Cancer Foundation (MCF)-7 cells under sodium valproate (2.0 mmol/L) for 24 h, 48 h, and 72 h. **B**. Cell apoptosis ratios were increased in a sodium valproate time- and concentration-dependent manner.

Cell apoptosis was analyzed using flow cytometry after different concentrations of sodium valproate (0.5-4.0 mmol/L) were added into the culture medium of MCF-7 cells. In the control group, the apoptosis showed no statistical changes with the extension of cell culture time, but different levels of increased apoptosis could be found under sodium valproate (Figure 2). For example, the apoptosis in the control cell group after 24 h, 48 h, and 72 h was 0.83 ± 0.19%, 1.62 ± 0.35%, and 2.76 ± 0.25%, respectively, whereas the apoptosis in the cell group exposed to 2.0 mmol/L sodium valproate for 24 h, 48 h, and 72 h was 4.61 ± 0.47%, 8.32 ± 0.75%, and 11.74 ± 0.95%, respectively (Figure 2A). There were also general changes in cell apoptosis under different concentrations of sodium valproate for 24 h, 48 h, and 72 h (Figure 2B). Pearson correlation analysis showed that the apoptosis of MCF-7 was positively correlated with sodium valproate concentrations (r = 0.925, *P* < 0.001).

### The G1 phase of cells cycle was arrested under VPA exposure

**Figure 3:**
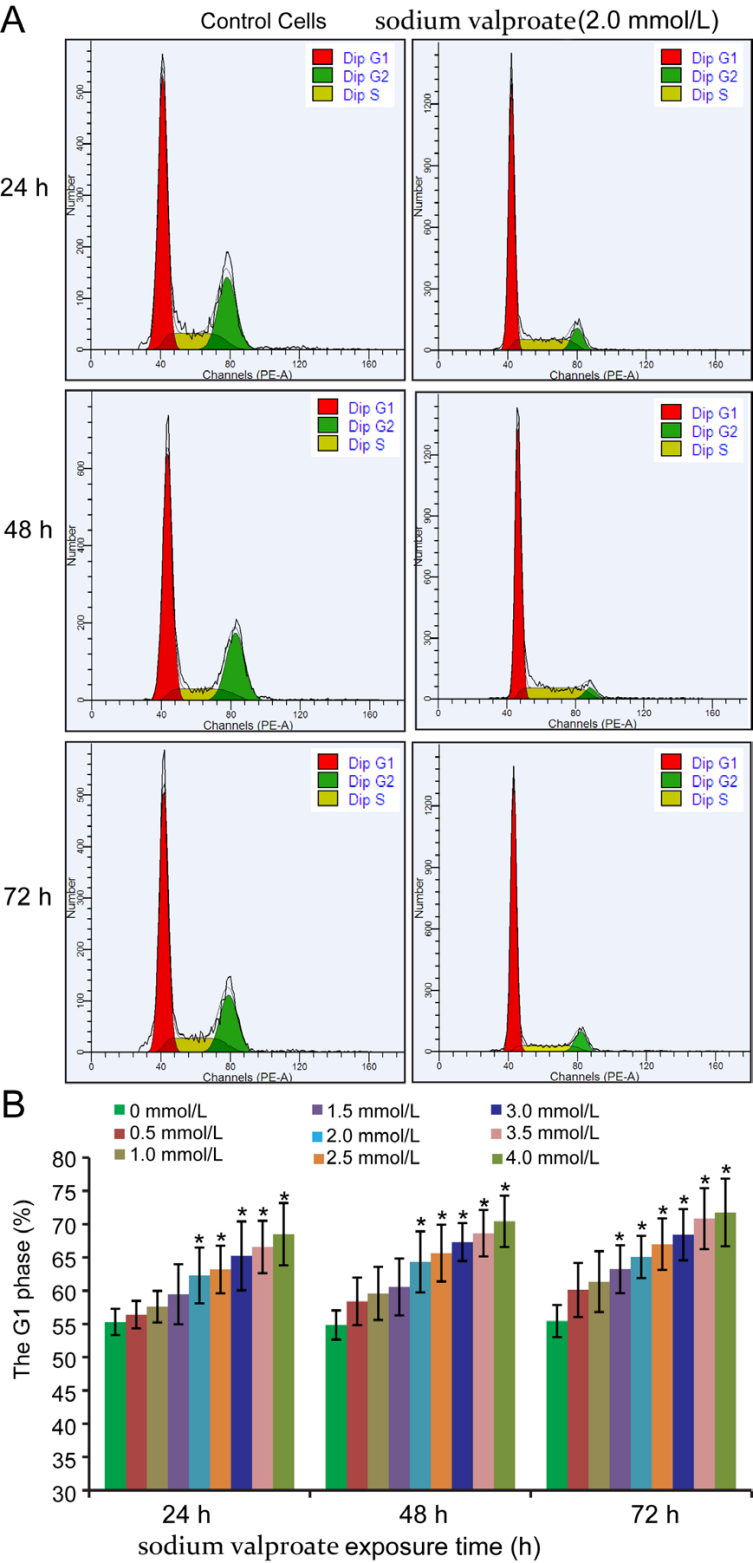
Cell cycle of Michigan Cancer Foundation (MCF)-7 was arrested in G1 phase under valproic acid (VPA) exposure. **A.** G1 phase of MCF-7 cells was arrested obviously under sodium valproate (2.0 mmol/L) for 24 h, 48 h, and 72 h. **B**. G1 phase was arrested in a VPA time- and concentration-dependent manner.

In cultured MCF-7 control cells, G1 phase, S phase, and G2 phase could be detected successively without obvious changes in the ratios of each phase with the extension of cell culture time. In sodium valproate-exposed cells, G1 phase was arrested at different degrees (Figure 3). The means of G1 phase in control cells cultured for 24 h, 48 h, and 72 h were 55.3 ± 1.98%, 54.86 ± 2.19%, and 55.44 ± 2.42%, respectively (Figure 3A). In cells exposed to 2.0 mmol/L sodium valproate, the median G1 phases after 24 h, 48 h, and 72 h were 62.3 ± 4.18%, 64.35 ± 4.57%, and 65.07 ± 3.19%, ie, statistically higher than that of control (*P* < 0.05). All changes of G1 phase in different sodium valproate-exposed cell groups are presented in Figure 3B. For cells exposed to 2.0 mmol/L sodium valproate for 24 h and 48 h, statistically significant G1 arrest could be observed (*P* < 0.05), as well as for cells exposed to sodium valproate 1.5 mmol/L (*P* < 0.05).

### Caspase-9 and caspase-3 involved in the VPA-induced apoptosis

**Figure 4:**
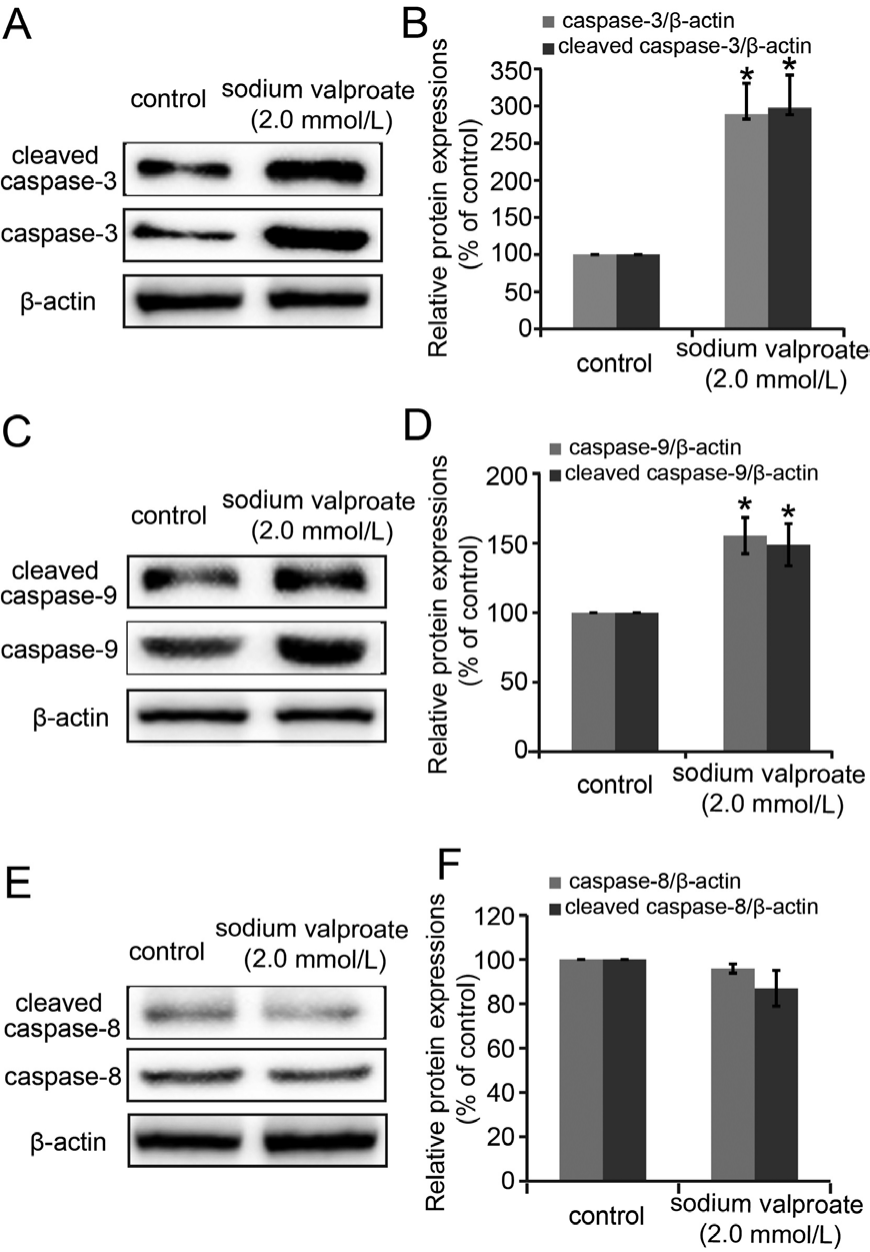
The changes of activity and protein expression levels of caspase-3, caspase-8, and caspase-9 under sodium valproate (2.0 mmol/L, 48 h) were detected. **A/B.** The changes and semi-quantification of caspase-3 and cleaved caspase-3. **C/D.** The changes and semi-quantification of caspase-9 and cleaved caspase-9. **E/F.** The changes and semi-quantification of caspase-8 and cleaved caspase-8.

To explore the molecular mechanism involved in VPA-induced MCF-7 cells apoptosis, we further investigated the activity and protein expressions of caspase-3, caspase-8, and caspase-9 under sodium valproate (2.0 mmol/L, 48 h) based on Western blot analysis (Figure 4). Both caspase-3 and cleaved caspase-3 (Figures 4A and 4B), caspase-9 and cleaved caspase-9 (Figures 4C and 4D) were significantly up-regulated in comparison wiht control (*P* < 0.05). Taking the relative protein expressions of control group as 100%, the levels of caspase-3, cleaved caspase-3, caspase-9, and cleaved caspase-9 were 289.20 ± 24.02%, 297.70 ± 26.75%, 155.34 ± 12.99%, and 148.49 ± 15.13%, respectively. However, for caspase-8 expression, no significant change was observed in sodium valproate-exposed cell group (Figures 4E and 4F).

### mRNA and protein expressions of cell cycle-related proteins

**Figure 5:**
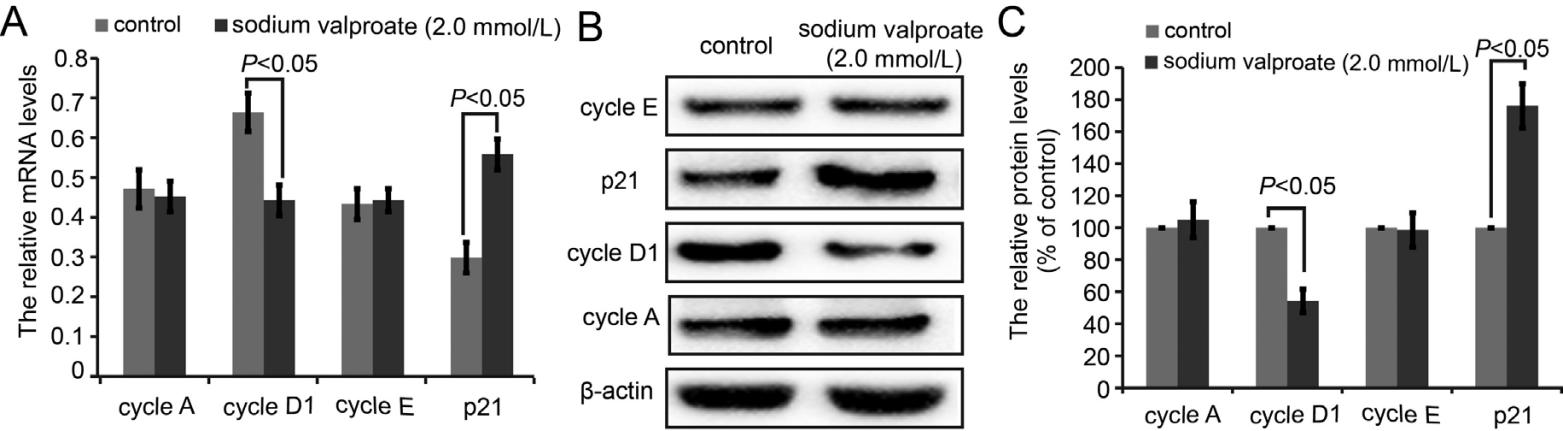
Analysis of cell cycle-related proteins, including cyclin A, cyclin D1, cyclin E, and p21. **A.** The mRNA levels of cyclin A, cyclin D1, cyclin E, and p21. **B.** The protein expressions of cyclin A, cyclin D1, cyclin E, and p21.**C.** The semi-quantifications of cyclin A, cyclin D1, cyclin E, and p21 protein expression based on Image J software.

The mRNA and protein expression levels of cell cycle-related proteins, including cyclin A, cyclin D1, cyclin E, and p21, were analyzed by RT-PCR and Western blot (Figure 5). After sodium valproate (2.0 mmol/L, 48 h) treatment, the mRNA levels of cyclin D1 decreased and p21 significantly increased (*P* < 0.05), but no significant changes were found in cyclin A and cyclin E levels (Figure 5A). For protein expressions, both cyclin D1 and p21 showed similar changes to mRNA, with cyclin D1 being down-regulated and p21 up-regulated. Cyclin A and cyclin E protein levels showed no statistically significant changes (Figures 5B and 5C).

## DISCUSSION

The present study showed that VPA, as a class I selective histone deacetylase inhibitor, suppressed the growth of breast cancer MCF-7 cells, induced apoptosis, and arrested G1 phase of MCF-7 cells. Intrinsic apoptotic pathway, marked as caspase-3 and caspase-9 activity elevation, was responsible for VPA-induced apoptosis. Further G1 phase arrest, p21 up-regulation, and cyclin D1 down-regulation may be the main molecules involved. Our data provided the theoretical support for VPA as an antitumor drug in breast cancer treatment.

The existing studies provided evidence that a HDAC inhibitor could suppress tumor cell proliferation, mainly reflected in inhibition of cell growth vigor (21), apoptosis induction (22), cell proliferation cycle arrest (23), and tumor cell re-differentiation induction (24). In the present study, we explored the role of sodium valproate on breast cancer MCF-7 cells, including cell growth, apoptosis, and proliferation. By applications of different concentrations of sodium valproate, we determined the decrease in cell viabilities, which suggested the sodium valproate-induced inhibition of MCF-7 cell growth. Our results showed that the cell viability changed in a sodium valproate time- and concentration-dependent manner. Additionally, the concentration for VPA used in this study was in mM range, which is reported to induce hepatotoxicity in cultured HepG2 by leading to mitochondrial respiration dysfunction (25). However, for antitumor function, 0-350 µM concentration of the VPA compounds was not reported to induce any significant inhibition of MCF-7 cells, which indirectly indicated that the VPA level needed for in MCF-7 cells inhibition had to be in mM range (15). The concentration used on Hela cells in that study was also in mM range (15). Therefore, we inferred that mM level of VPA was needed for anti-breast cancer effect. However, this concentration adversely affects the liver function.

For cell apoptosis detection, we used low cytometry to determine the apoptosis ratios based on a series of different sodium valproate concentrations. The apoptotic rates obviously increased with increasing sodium valproate doses, which confirmed the induction of apoptosis by sodium valproate. Further, we explored the molecular mechanism involved in sodium valproate-induced apoptosis. Caspase was the central component of apoptosis, and caspase cascade was the actuator of apoptosis. Based on its role in apoptosis, caspase family was divided into the starting-type caspases (such as caspase-8 and caspase 9) and the effect-type caspases (such as caspase-1, caspase-3, caspase-6, and caspase-7). The activation of effect-type caspase acted on the respective substrates, inducing apoptosis characterized as DNA fragmentation at the nucleosome. According to the different component of caspase, cell apoptosis pathway was divided into extracellular pathway (death receptor pathway) and intrinsic apoptotic pathway (mitochondrial pathway) (26). Caspase-9 and caspase-3 are two important molecular in intrinsic apoptotic pathway and induce a series of downstream events during apoptosis, whereas caspase-8 is the main molecule involved in the extracellular pathway of apoptosis (27). Thus, we investigated the protein expressions of caspase-9, caspase-3, and caspase-8 during VPA exposure. Our results showed that sodium valproate exposure increased caspase-3 and caspase-9 activity and protein levels, but not caspase-8 activity. This suggested that VPA induced MCF-7 apoptosis mainly through the intrinsic apoptotic pathway, but not through the extracellular pathway. Similar findings were reported in another study (28). For example, N-hydroxy-4-(4-phenylbutyryl-amino) benzamide (HTPB), as a novel HDACi, was shown as a potential chemotherapeutic agent in lung cancer (29). It significantly suppressed lung cancer cells’ proliferation and metastases and induced mitochondria-mediated apoptosis. A nanoformulation of doxorubicin, Doxil, was believed to be active against breast and ovarian cancers (30). Doxil exposure of human breast adenocarcinoma cells MCF-7 inhibited the activity of HDAC and enhanced apoptosis with a significant increase in the loss of mitochondrial membrane potential, DNA fragmentation percentage, and so on. Tumor is a cell cycle-related disease and there are many regulatory points, with G1/S conversion and G2/M conversion as the most important (31). Tumor cells can smoothly pass the G1/S transition and G2/M transition points, which results in unlimited cell proliferation. We observed the cell cycle distributions under different concentrations of VPA and found an increased percentage of cells in G1 phase under sodium valproate treatment, which was consistent with other reports for human neuroblastoma BE (2)-C cells (32) and neuronal cells (33).

Additionally, the transcription and protein expression of cyclins could be blocked by cyclin-dependent kinase (CDK) inhibitors. Seven types of CDK inhibitors belonging to two families, INK4 and CIP/KIP, have been reported. CIP/KIP family, also known as p21 family including p21, p27, and p57, could inhibit the role of cyclin-CDK (34). Our assay results of the mRNA and protein levels of cyclin A, cyclin D1, cyclin E, and p21 showed the up-regulation of p21 and down-regulation of cyclin D1, providing some clues for the molecular mechanism of VPA to G1 phase arrest.

Although some molecular mechanism have been indicated in this study, further research is needed to elucidate the many details involved in this possible pathway and the underlying molecular mechanism of VPA in breast cancer treatment.

In summary, our results indicate that VPA’s effect on MCF-7 cells includes inhibit cell growth, apoptosis induction, and G1 phrase arrest. The mechanism underlying the effect on apoptosis induction involves the intracellular pathways (mitochondrial pathway). The mechanism underlying VPA effect on the cell cycle arrest in G1 phrase induction include up-regulation of p21 mRNA and protein expression, which can bind CDKs competitively with cyclins, thus reducing the cyclin-CDK complexes and inducing G1 phrase arrest. Due to down-regulation of cyclin D1, mRNA, and protein expression inhibiting cyclin D1-CDK4/CDK6 pathway activity, cells cannot pass through the G1/S check point.

## References

[1] Sincennes MC, Brun CE, Rudnicki MA (2016). Concisereview: epigenetic regulation of myogenesis in health and disease.. Stem Cells Transl Med.

[2] Chen HP, Zhao YT, Zhao TC (2015). Histone deacetylases and mechanisms of regulation of gene expression.. Crit Rev Oncog.

[3] Haery L, Thompson RC, Gilmore TD (2015). Histone acetyltransferases and histone deacetylases in B- and T-cell development, physiology and malignancy.. Genes Cancer.

[4] Yang H, Salz T, Zajac-Kaye M, Liao D, Huang S, Qiu Y (2014). Overexpression of histone deacetylases in cancer cells is controlled by interplay of transcription factors and epigenetic modulators.. FASEB J.

[5] Pang M, Zhuang S (2010). Histone deacetylase: a potential therapeutic target for fibrotic disorders.. J Pharmacol Exp Ther.

[6] Xie C, Wu B, Chen B, Shi Q, Guo J, Fan Z (2016). Histone deacetylase inhibitor sodium butyrate suppresses proliferation and promotes apoptosis in osteosarcoma cells by regulation of the MDM2-p53 signaling.. Onco Targets Ther.

[7] Brazelle W, Kreahling JM, Gemmer J, Ma Y, Cress WD, Haura E (2010). Histone deacetylase inhibitors downregulate checkpoint kinase 1 expression to induce cell death in non-small cell lung cancer cells.. PLoS One.

[8] Hrgovic I, Doll M, Kleemann J, Wang XF, Zoeller N, Pinter A (2016). The histone deacetylase inhibitor trichostatin a decreases lymphangiogenesis by inducing apoptosis and cell cycle arrest via p21-dependent pathways.. BMC Cancer.

[9] Nagańska E, Matyja E, Taraszewska A, Rafałowska J (2015). Protective effect of valproic acid on cultured motor neurons under glutamate excitotoxic conditions. Ultrastructural study.. Folia Neuropathol.

[10] Göttlicher M, Minucci S, Zhu P, Kramer OH, Schimpf A, Giavara S (2001). Valproic acid defines a novel class of HDAC inhibitors inducing differentiation of transformed cells.. EMBO J.

[11] Kwiecińska P, Wiśniewska J, Gregoraszczuk EŁ (2011). Effects of valproic acid (VPA) and levetiracetam (LEV) on proliferation, apoptosis and hormone secretion of the human choriocarcinoma BeWo cell line.. Pharmacol Rep.

[12] Xu Y, Xu D, Zhu SJ, Ye B, Dong JD, Zhang YL (2015). Induction of apoptosis and autophagy in metastatic thyroid cancer cells by valproic acid (VPA).. Int J Clin Exp Pathol.

[13] Li X, Zhu Y, He H, Lou L, Ye W, Chen Y (2013). Synergistically killing activity of aspirin and histone deacetylase inhibitor valproic acid (VPA) on hepatocellular cancer cells.. Biochem Biophys Res Commun.

[14] Gilardini Montani MS, Granato M, Santoni C, Del Porto P, Merendino N, D’Orazi G (2017). Histone deacetylase inhibitors VPA and TSA induce apoptosis and autophagy in pancreatic cancer cells.. Cell Oncol (Dordr).

[15] Prestegui-Martel B, Bermúdez-Lugo JA, Chávez-Blanco A, Dueńas-González A, García-Sánchez JR, Pérez-González OA (2016). N-(2-hydroxyphenyl)-2-propylpentanamide, a valproic acid aryl derivative designed in silico with improved anti-proliferative activity in HeLa, rhabdomyosarcoma and breast cancer cells.. J Enzyme Inhib Med Chem.

[16] Reid G, Métivier R, Lin CY, Denger S, Ibberson D, Ivacevic T (2005). Multiple mechanisms induce transcriptional silencing of subset of genes, including oestrogenreceptor alpha, in response to deacetylase inhibition by valproic acid and trichostatin A.. Oncogene.

[17] Sang Z, Sun Y, Ruan H, Cheng Y, Ding X, Yu Y (2016). Anticancer effects of valproic acid on oral squamous cell carcinoma via SUMOylation in vivo and in vitro.. Exp Ther Med.

[18] Terranova-Barberio M, Roca MS, Zotti AI, Leone A, Bruzzese F, Vitagliano C (2016). Valproic acid potentiates the anticancer activity of capecitabine in vitro and in vivo in breast cancer models via induction of thymidine phosphorylase expression.. Oncotarget.

[19] Fortunati N, Bertino S, Costantino L, Bosco O, Vercellinatto I, Catalano MG (2008). Valproic acid is a selective antiproliferative agent in estrogen-sensitive breast cancer cells.. Cancer Lett.

[20] Yang W, Zhao X, Pei F, Ji M, Ma W, Wang Y (2015). Activation of the intrinsic apoptosis pathway contributes to the induction of apoptosis in hepatocellular carcinoma cells by valproic acid.. Oncol Lett.

[21] Garrett LA, Growdon WB, Rueda BR, Foster R (2016). Influence of a novel histone deacetylase inhibitor panobinostat (LBH589) on the growth of ovarian cancer.. J Ovarian Res.

[22] Park KC, Heo JH, Jeon JY, Choi HJ, Jo AR, Kim SW (2015). The novel histone deacetylase inhibitor, N-hydroxy-7-(2-naphthylthio) hepatonomide, exhibits potent antitumor activity due to cytochrome-c-release-mediated apoptosis in renal cell carcinoma cells.. BMC Cancer.

[23] Mawatari T, Ninomiya I, Inokuchi M, Harada S, Hayashi H, Oyama K (2015). Valproic acid inhibits proliferation of HER2-expressing breast cancer cells by inducing cell cyclearrest and apoptosis through Hsp70 acetylation.. Int J Oncol.

[24] Blagitko-Dorfs N, Jiang Y, Duque-Afonso J, Hiller J, Yalcin A, Greve G (2013). Epigenetic priming of AML blasts for all-trans retinoic acid-induced differentiation by the HDAC class-I selective inhibitor entinostat.. PLoS One.

[25] Komulainen T, Lodge T, Hinttala R, Bolszak M, Pietilä M, Koivunen P (2015). Sodium valproate induces mitochondrial respiration dysfunction in HepG2 in vitro cell model.. Toxicology.

[26] Cagnol S, Chambard JC (2010). ERK and cell death: mechanisms of ERK-induced cell death–apoptosis, autophagy and senescence.. FEBS J.

[27] Hajra KM, Liu JR (2004). Apoptosome dysfunction in human cancer.. Apoptosis.

[28] Singh TR, Shankar S, Srivastava RK (2005). HDAC inhibitors enhance the apoptosis-inducing potential of TRAIL in breast carcinoma.. Oncogene.

[29] Shieh JM, Wei TT, Tang YA, Huang SM, Wen WL, Chen MY (2012). Mitochondrial apoptosis and FAK signaling disruption by a novel histone deacetylase inhibitor, HTPB, in antitumor and antimetastatic mouse models.. PLoS One.

[30] Zakaria S, Gamal-Eldeen AM, El-Daly SM, Saleh S (2014). Synergistic apoptotic effect of Doxil® and aminolevulinic acid-based photodynamic therapy on human breast adenocarcinoma cells.. Photodiagnosis Photodyn Ther.

[31] Singh RP, Agarwal R (2006). Natural flavonoids targeting deregulated cell cycle progression in cancer cells.. Curr Drug Targets.

[32] Cinatl J, Kotchetkov R, Blaheta R, Driever PH, Vogel JU, Cinatl J (2002). Induction of differentiation and suppression of malignant phenotype of human neuroblastoma BE (2)-C cells by valproic acid: enhancement by combination with interferon α.. Int J Oncol.

[33] Bacon CL, Gallagher HC, Haughey JC, Regan CM (2002). Antiproliferative action of valproate is associated with aberrant expression and nuclear translocation of cyclin D3 during the C6 glioma G1 phase.. J Neurochem.

[34] Park HY, Kim MK, Moon SI, Cho YH, Lee CH (2006). Cell cycle arrest and apoptotic induction in LNCaP cells by MCS-C2, novel cyclin-dependent kinase inhibitor, through p53/p21WAF1/CIP1 pathway.. Cancer Sci.

